# Remote cooling by a novel thermal lens with anisotropic positive thermal conductivity

**DOI:** 10.1038/srep40949

**Published:** 2017-01-18

**Authors:** Fei Sun, Sailing He

**Affiliations:** 1 Centre for Optical and Electromagnetic Research, Zhejiang Provincial Key Laboratory for Sensing Technologies, JORCEP, East Building #5, Zhejiang University, Hangzhou 310058, China; 2 Department of Electromagnetic Engineering, School of Electrical Engineering, Royal Institute of Technology (KTH), S-100 44 Stockholm, Sweden

## Abstract

A novel thermal lens that can achieve a remote cooling effect is designed by transformation thermodynamics. The effective distance between the separate hot source and cold source is shortened by our shelled thermal lens without any negative thermal conductivity. Numerical simulations verify the performance of our thermal lens. Based on the effective medium theory, we also propose a practical way to realize our lens using two-layered isotropic thermal media that are both found in nature. The proposed thermal lens will have potential applications in remote temperature control and in creating other thermal illusions.

Transformation Optics (TO) can be utilized to design special electromagnetic media with pre-designed functions by using a coordinate transformation from the virtual space to the real space^1^,^2^,^3^. Many novel optical/electromagnetic devices have been designed by TO, including invisibility cloakings^4^,^5^,^6^, novel lenses^7^,^8^, field rotators^9^,^10^,^11^, concentrators^12^, illusion optical devices^13^,^14^, and others. Based on the heat equation’s form-invariant property under coordinate transformations, transformation thermodynamics (TT) was first proposed by Guenneau, S. *et al*.^15^. In recent years, manipulating heat flux by a coordinate transformation method has attracted much attention and developed many novel thermal devices, e.g. thermal cloakings^16^,^17^,^18^, concentrators for heat collections^19^,^20^, heat hyper-lenses for heat focusing^21^, heat hoses^22^, heat flux rotators^23^, thermal diodes^24^, heat illusion devices^25^,^26^,^27^,^28^,^29^, novel thermal lenses^30^,^31^, devices for a heat directional transmission^32^, etc.

In 2016, our group proposed a novel thermal lens based on spatial translations and folding coordinate transformations together to achieve a remote heating/cooling operation on temperature distribution^31^. However, it is still challenging to realize the proposed thermal lens due to the negative thermal conductivity introduced from the spatial folding transformation. In some applications, we need a remote cooling technique. In some applications, we want to achieve a very high temperature in an given region (the target region) while not influencing its neighboring area (i.e. the temperature is low around the target region). We need to use a remote cooling technique to cool down the temperature around the target region but not influencing the hot target region. For example, in laser tumor therapy, we hope normal cells around the targeted tumor cell are not killed by the high temperature produced by the laser. In this case, we need a cooling technique to keep the temperature in normal cells relatively low. The cooling source cannot be set inside the human body, which means that a remote cooling technique is required. Such a remote cooling can also be applied to cool down the neighboring area around an inevitable hot site through a remote cold source. In this study, we use the spatial translation and compression transformations together to achieve a remote cooling effect using a thermal lens with homogeneous positive anisotropic thermal conductivity.

## Methods

**Figure 1:**
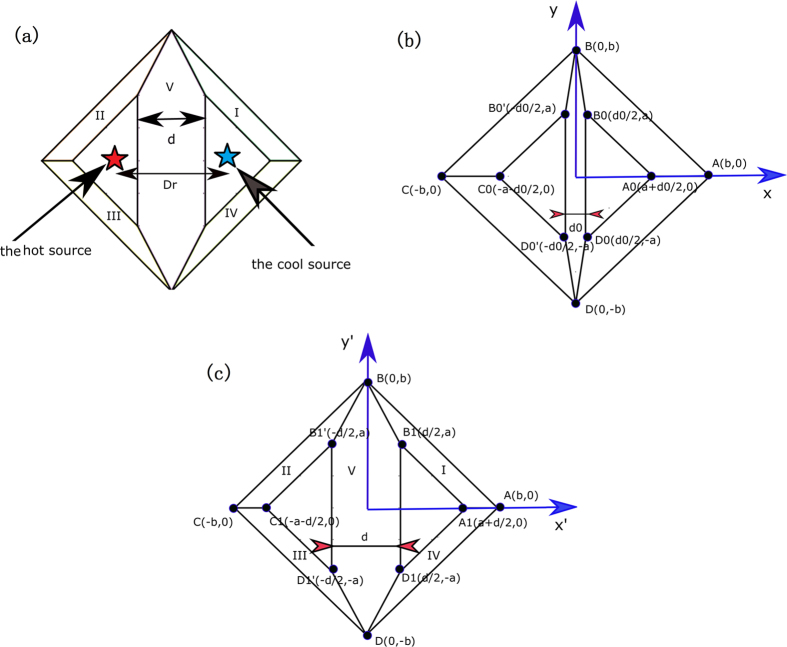
(**a**) The basic structure of our thermal lens. Five regions (labeled by I, II, III, IV and V) comprise our thermal lens with homogenous anisotropic thermal media. All other regions are the background medium. The hot source (red star) and cool source (blue star) are set in the background medium. The coordinate transformation between the reference space (**b**) and the real space (**c**). In (**c**), quadrangles *BB*_1_*A*_1_*A, BB*_1_′*C*_1_*C, DD*_1_′*C*_1_*C*, and *DD*_1_*A*_1_*A* correspond to regions I, II, III and IV. The polygon *BB*_1_*D*_1_*DD*_1_′*B*_1_′ corresponds to region V.

Figure 1(a) shows the basic function of our thermal lens. The whole lens is composed of five different regions of media with homogenous anisotropic thermal conductivity (indicated by different labels). The hot source (a red star) and cool source (blue star) are set in the left and right sides of the region V, respectively, within the shelled structure (i.e. our lens). The function of this shelled lens is to reduce the effective spatial distance between the hot source and the cool source, which makes the cool source effectively much closer to the hot source than their real distance. The effective cooling region is the region outside the shelled structure *ABCD*.

We now show how to design the proposed lens using TT. Figure 1(b) and (c) show the corresponding relation between the reference space and the real space. The quantities with and without primes are those in the real space and the reference space, respectively, throughout the paper. Two triangular regions, *B*_1_*A*_1_*D*_1_ and *B*_1_′*C*_1_*D*_1_′, in real space are spatially translated from *B*_0_*A*_0_*D*_0_ and *B*_0_′*C*_0_*D*_0_′, respectively, in the reference space. The thermal materials in these two regions are still the same as the background medium due to the spatial translational transformation. The middle region (i.e. Region V) and the shelled regions (i.e. regions I–IV) are compressed and stretched, respectively, from the real space to the reference space. The whole coordinate transformation can be summarized as:


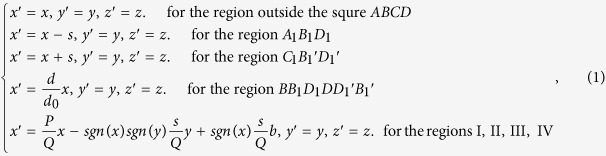


where 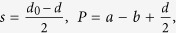 and 
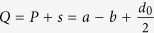
. As shown in Fig. 1(c), *a, b* and *d* determine the geometrical size of our shell in the real space. Note that *sgn* is the sign function, which is defined by:


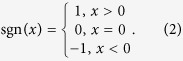


The thermal conductivity in each region can be calculated with the help of TT^15^. Only the regions I–V in Fig. 1(a) or (c) have thermal conductivity different from the background medium (i.e. other regions without labels) with a constant isotropic thermal conductivity *κ*_0_. The required thermal conductivity in the region V is given by:


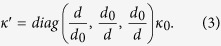


The required thermal conductivity in the other four regions I–IV can be summarized by:


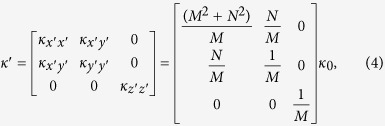


where 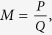 and 
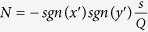
. The distance between the two triangular regions *B*_1_*A*_1_*D*_1_ and *B*_1_′*C*_1_*D*_1_′ in the real space is *d*, which is also the thickness of the region V. The function of the whole coordinate transformation (i.e. the effect of the transformation medium) is to shorten the effective distance between these two regions. Actually, the effective distance between these two regions is *d*_0_ if the other regions I–V are filled by our lens described by Eqs (3) and (4). Since the effective distance between the two triangular regions is shortened (e.g. *d*_0_ « *d*), the cooling effect would be improved if we set a hot source and a cold source at any position in these two triangular regions, separately.

We use *D*_*r*_ to indicate the real distance between the hot and cold sources, which includes the distance from the two sources to the edge of region V and the physical thickness of region V (see Fig. 1(a)). Since the function of our lens is to shorten the effective distance between the two triangular regions *B*_1_*A*_1_*D*_1_ and *B*_1_′*C*_1_*D*_1_′ (i.e. the thickness of region V), the effective distance between the hot and cold sources are *D*_*e*_ = *D*_*r*_ − *d* + *d*_*0*_.

## Results

**Figure 2:**
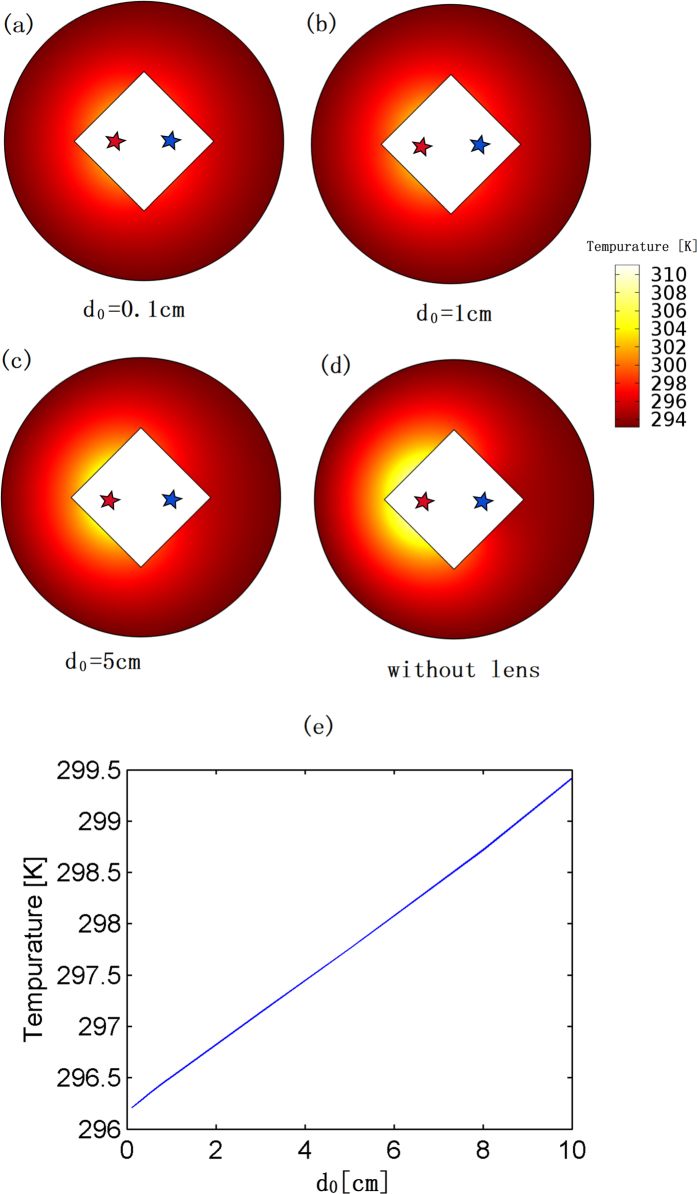
The finite element method (FEM) simulation results. We plot the temperature distribution outside our thermal lens (colored white): (**a**)–(**c**) with our thermal lens, and (**d**) without our thermal lens. The outer boundary is set by the room temperature (295 *K*). We set one hot point source with fixed temperature 375 *K* and a cold point source with fixed temperature 275 *K* inside our thermal lens (marked by red and blue stars, respectively). The background medium is chosen as a thermal epoxy with a conductivity of 3.4 *W/(m·K*), a mass density of 3.1e^3^ *kg*/*m*^3^, and a thermal capacity of 550 *J*/(*kg·K*). The conductivity of our thermal lens is given by Eqs (3) and (4) with *d* = 10 *cm, a* = 10 *cm*, and *b* = 20 *cm*. From (**a**) to (**c**), *d*_0_ changes from 0.1 *cm* to 5 *cm,* as marked below each figure. The real distance between the hot source and the cold source is fixed at *D*_*r*_ = 12 *cm*. (**e**) The relation between the temperature at a fixed point outside the lens (i.e. 20 *cm* away from the hot source) and the parameter *d*_0_. As *d*_0_ becomes smaller, the temperature of the fixed point becomes lower (i.e. it approaches 295 *K*, the fixed temperature of the boundary of the whole calculation domain), which indicates a better cooling effect with the lens.

Numerical simulations are given for a hot point source (375 *K*) and a cold point source (275 *K*) separated by a fixed distance *D*_*r*_ = 12 *cm* with and without our lens, respectively, in Fig. 2(a) and (d). The temperature distribution outside our lens is much lower than the case in which the lens is removed, which means that the cooling effect is greatly improved by introducing our lens.

We could expect that if *d*_0_ is smaller, the effective spatial distance between the hot source and the cold source is smaller (*D*_*e*_ = *D*_*r*_ − *d* + *d*_*0*_), which indicates a better cooling effect. As shown in Fig. 2, the cooling effect improves as *d*_0_ becomes smaller while keeping the physical distance between the hot source and the cold source *D*_*r*_ unchanged.

**Figure 3:**
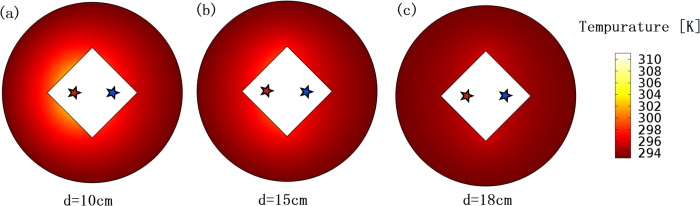
The finite element method (FEM) simulation results. We plot the temperature distribution outside our thermal lens (colored white): (**a**)–(**c**), *d* changes from 10 *cm* to 18 *cm* as marked below each figure. We keep other parameters unchanged *d*_0_ = 1 *cm, a* = 10 *cm*, and *b* = 20 *cm*. The real distance between the hot source and the cold source is unchanged (i.e. *D*_*r*_ = 12 *cm*). Other settings are the same as in Fig. 2.

Another interesting aspect is that, if the real distance between the hot source and the cold source is unchanged, and only the parameter *d* in Eq. (4), which determines the thermal media of our thermal lens, is changed (other parameters *a, b, d*_0_ do not change), the performance of our thermal lens also changes. Considering the effective distance between the hot and cold sources are *D*_*e*_ = *D*_*r*_ − *d* + *d*_*0*_, and thus the larger *d*, the better cooling effect (see Fig. 3). Note that *d* cannot be too much larger, given that the geometrical size of the whole lens is fixed (i.e. *d*/2 + *a* < *b*).

### Experiment design

Since thermal conductivity in each region of the whole lens is positive, it can be realized by naturally-available materials. We design a feasible thermal lens with layered isotropic thermal materials based on the effective medium theory. The background medium is chosen to be thermal epoxy with a constant thermal conductivity of 3.4 *W/(m·K*). The size of the lens is chosen as *d* = 20 *cm, d*_0_ = 1 *cm, a* = 5 *cm, b* = 20 *cm*. We use two layered isotropic thermal media to achieve an effective anisotropic thermal conductivity:


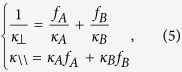


where *κ*_A_ and *κ*_B_ are thermal conductivities of two isotropic media. *κ*_\\_ and *κ*_⊥_ are the effective anisotropic thermal conductivities along and orthogonal to the direction of interface between two isotropic media, respectively. *f*_*A*_ and *f*_*B*_ are the filling factor of the medium A and the medium B, respectively, which satisfy *f*_*A*_ + *f*_*B*_ = 1.

For the region V in the middle of our device, we choose copper with thermal conductivity of 394 *W/(m·K*) as the medium A. *κ*_\\_ and *κ*_*⊥*_ are determined by parameters *d* and *d*_0_ (i.e. the real distance and the effective distance between the hot source and the cold source, respectively), and calculated by Eq. (3): *κ*_\\_ = 68 and *κ*_*⊥*_ = 0.17. The required filling factor of the medium A and the thermal conductivity of the medium B are two unknown quantities which can be solved from Eq. (5): *f*_*A*_ = 17.2% and *κ*_B_ = 0.141 *W/(m·K*). We can use PVC with thermal conductivity of 0.14 *W/(m·K*) as the medium B.

**Figure 4:**
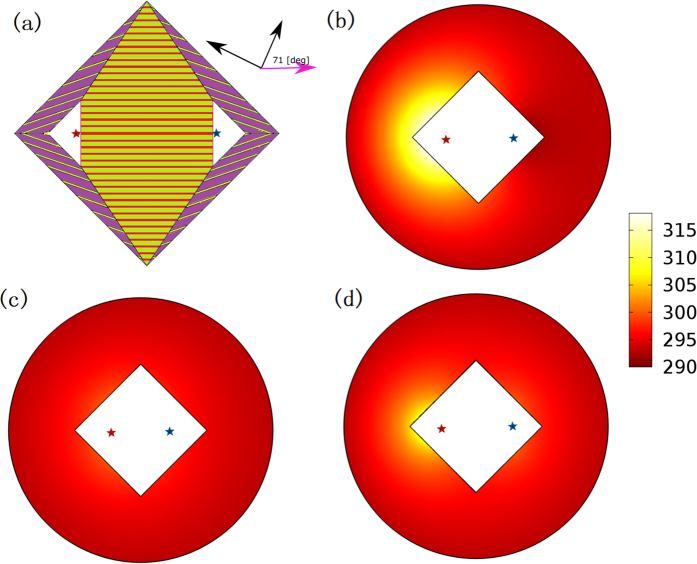
(**a**) The structure of the thermal lens composed of layered media. In the middle region, two media are copper (colored red) and PVC (colored green). In other four regions, the two media are bismuth (colored yellow) and fused silica (colored purple). (**b**)–(**d**) are numerical simulation results by finite element method. We plot the temperature distribution outside our lens. (**c**) the theoretical lens without layering and (**d**) the lens composed of the layered media in (**a**). (**b**) is the case in which the lens is removed. The red star and blue star indicate the locations of the hot source and the cold source, respectively. All other settings are consistent with Fig. 2.

For the Regions I, II, III and IV, we only need to design two layered-media in Region I, and media in other regions can be obtained by the symmetry transformation due to the symmetry of the whole lens with respect to the *x*′ and *y*′ axes (see the structure in Fig. 4(a)). The direction of the principal axis of the anisotropic medium in Region I can be obtained by diagonalizing the matrix in Eq. (4) 33:


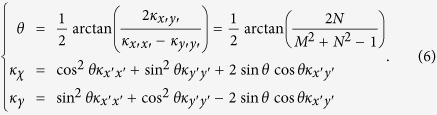


*χ*-*γ* system is the local principal axis system, in which the anisotropic thermal conductivity in Eq. (4) can be expressed by a diagonal matrix. For the thermal lens with the same parameters as in Fig. 2, the angle between the *x*′-axis and *χ*-axis is *θ* = 71[deg], and the two principal values of thermal conductivity are 1.6 *W/(m·K*) and 2.9 *W/(m·K*) (i.e. *κ*_\\_ = 2.9 and *κ*_⊥_ = 1.6). We choose bismuth, with thermal conductivity of nearly 8 *W/mK,* as medium A. By solving Eq. (5), we can obtain the required thermal conductivity of the medium B and the filling factor: *κ*_B_ = 1.275 *W/(m·K*) and *f*_*A*_ = 24.2%. Thus, fused silica with thermal conductivity of 1.32 *W/(m·K*) can be used as medium B^34^. The structure of the lens is shown in Fig. 4(a). Numerical simulation results of the lens composed of a two-layered medium are given in Fig. 4(d), which shows good cooling effect compared to the case where the lens is removed in Fig. 4(b).

We should note that the purpose of our design is to cool down the region outside the shell *ABCD* in Fig. 1(c) by our lens. We only plot the temperature distribution outside the shell in all our simulations (e.g. in Figs 2, 3 and 4). If we add the temperature distribution of other regions (e.g. inside our lens), we have to rescale the colorbar of the figures and consequently it will be hard to see the difference of the temperature distribution outside the shell for the cases with and without the lens from the figures.

## Conclusion

By combining spatial translations and compression transformations, we designed a novel thermal lens for a remote cooling function. The hot source and the cold source can be separated by a certain distance while keeping a good cooling effect by applying our thermal lens around them. Based on the effective medium theory, we also design a specific lens composed of a layered isotropic thermal medium that is available in nature. Numerical simulations verify the function of the proposed lens.

## Additional Information

**How to cite this article:** Sun, F. and He, S. Remote cooling by a novel thermal lens with anisotropic positive thermal conductivity. *Sci. Rep.* **7**, 40949; doi: 10.1038/srep40949 (2017).

**Publisher's note:** Springer Nature remains neutral with regard to jurisdictional claims in published maps and institutional affiliations.
